# Efficient Pneumonia Detection in Chest Xray Images Using Deep Transfer Learning

**DOI:** 10.3390/diagnostics10060417

**Published:** 2020-06-19

**Authors:** Mohammad Farukh Hashmi, Satyarth Katiyar, Avinash G Keskar, Neeraj Dhanraj Bokde, Zong Woo Geem

**Affiliations:** 1 Department of Electronics and Communication Engineering, National Institute of Technology, Warangal 506004, India; mdfarukh@nitw.ac.in; 2 Department of Electronics and Communication Engineering, Harcourt Butler Technical University, Kanpur 208002, India; satyarth9@gmail.com; 3 Department of Electronics and Communication Engineering, Visvesvaraya National Institute of Technology, Nagpur 440010, India; agkeskar@ece.vnit.ac.in; 4 Department of Engineering-Renewable Energy and Thermodynamics, Aarhus University, 8000 Aarhus, Denmark; neerajdhanraj@eng.au.dk; 5 Department of Energy IT, Gachon University, Seongnam 13120, Korea

**Keywords:** pneumonia, chest X-ray images, convolution neural network (CNN), deep learning, transfer learning, computer-aided diagnostics

## Abstract

Pneumonia causes the death of around 700,000 children every year and affects 7% of the global population. Chest X-rays are primarily used for the diagnosis of this disease. However, even for a trained radiologist, it is a challenging task to examine chest X-rays. There is a need to improve the diagnosis accuracy. In this work, an efficient model for the detection of pneumonia trained on digital chest X-ray images is proposed, which could aid the radiologists in their decision making process. A novel approach based on a weighted classifier is introduced, which combines the weighted predictions from the state-of-the-art deep learning models such as ResNet18, Xception, InceptionV3, DenseNet121, and MobileNetV3 in an optimal way. This approach is a supervised learning approach in which the network predicts the result based on the quality of the dataset used. Transfer learning is used to fine-tune the deep learning models to obtain higher training and validation accuracy. Partial data augmentation techniques are employed to increase the training dataset in a balanced way. The proposed weighted classifier is able to outperform all the individual models. Finally, the model is evaluated, not only in terms of test accuracy, but also in the AUC score. The final proposed weighted classifier model is able to achieve a test accuracy of 98.43% and an AUC score of 99.76 on the unseen data from the Guangzhou Women and Children’s Medical Center pneumonia dataset. Hence, the proposed model can be used for a quick diagnosis of pneumonia and can aid the radiologists in the diagnosis process.

## 1. Introduction

Pneumonia is an acute respiratory infection that affects the lungs. It is a fatal illness in which the air sacs get filled with pus and other liquid [1]. There are mainly two types of pneumonia: bacterial and viral. Generally, it is observed that bacterial pneumonia causes more acute symptoms. The most significant difference between bacterial and viral pneumonia is the treatment. Treatment of bacterial pneumonia is done using antibiotic therapy, while viral pneumonia will usually get better on its own [2]. It is a prevalent disease all across the globe. Its principal cause includes a high level of pollution. Pneumonia is ranked eight in the list of the top 10 causes of death in the United States [3]. Due to pneumonia, every year, 3.7 lakh children die in India, which constitutes a total of fifty percent of the pneumonia deaths that occur in India [4]. The disease frequently goes overlooked and untreated until it has reached a fatal point, especially in the case of old patients. It is the single largest cause of death in children (especially under the age of five) worldwide [5]. According to the WHO, “Every year, it kills an estimated 1.4 million children under the age of five years, accounting for 18% of all deaths of children under five years old worldwide. Pneumonia affects children and families everywhere but is most prevalent in South Asia and sub-Saharan Africa. Children can be protected from pneumonia. It can be prevented with simple interventions and treated with low-cost, low-tech medication and care” [2]. Therefore, there is an urgent need to do research and development on computer-aided diagnosis so that the pneumonia-related mortality, especially in children, can be reduced.

One of the following tests can be done for pneumonia diagnosis: chest X-rays, CT of the lungs, ultrasound of the chest, needle biopsy of the lung, and MRI of the chest [6]. Currently, chest X-rays are one of the best methods for the detection of pneumonia [7]. X-ray imaging is preferred over CT imaging because CT imaging typically takes considerably more time than X-ray imaging, and sufficient high-quality CT scanners may not be available in many underdeveloped regions. In contrast, X-rays are the most common and widely available diagnostic imaging technique, playing a crucial role in clinical care and epidemiological studies [8,9]. There are several regions across the globe where there is a scarce availability of practiced healthcare workers and radiologists whose prediction on such diseases matter greatly [10,11,12]. Computer-aided diagnosis using artificial intelligence based solutions is becoming increasingly popular these days [13,14]. This facility can be made available to a large population at a minimal cost. Another issue with this disease is that sometimes, the features that describe the very existence of the disease often get mixed with other diseases, and hence, radiologists find it challenging to diagnose this disease. Deep learning techniques solve all these problems, and their accuracy in the prediction of the disease is the same and sometimes even greater than an average radiologist [15]. Among the deep learning techniques, convolutional neural networks (CNNs) have shown great promise in image classification and segmentation and therefore are widely adopted by the research community. Biomedical image diagnosis that uses the techniques of deep learning and computer vision has proven to be very helpful to provide a quick and accurate diagnosis of the disease that matches the accuracy of a reliable radiologist [16]. Currently, deep learning based methods cannot replace trained clinicians in medical diagnosis, and they aim to supplement clinical decision making. In this paper, a model is presented based on the applications of deep learning and convolutional neural networks that are capable of classifying automatically that the patient has pneumonia or not. The proposed methodology uses a deep transfer learning algorithm that extracts the features from the X-ray image that describes the presence of disease automatically and reports whether it is a case of pneumonia.

## 2. Related Work

Deep learning based methods are already being used in various fields [17,18,19,20,21]. Different authors have already proposed several biomedical image detection techniques. M.I.Razaak [22] discussed the challenges and the future of medical image processing. Much work has already been done for the detection of numerous diseases by using deep learning based techniques, as stated by Dinggang Shen [23]. Andre [24] presented a deep learning model for dermatologist-level classification of skin cancer, and F.Milletari [25] also proposed a methodology for the depiction of prostrate in MRI volumes using CNN. Grewal [26] used the technique of deep learning for brain hemorrhage detection in CT scans, and Varun [27] proposed a method for detecting diabetic retinopathy in retinal fundus photographs. Y. Bar [28] also discussed chest pathology detection by the techniques based on deep learning. Methods regarding the examination of the detection of disease by chest X-ray have also been worked on earlier by performing various examination techniques [29,30,31]. The chest X-ray images are passed through the evaluation process of scan line optimization such that it eliminates all the other body parts to avoid any error in diagnosis. The algorithm was described by S. Hermann [32]. Nasrullah et al. [33] used two deep three-dimensional (3D) customized mixed link network (CMixNet) architectures for lung nodule detection and classification. Yao L et al. [34] combined DenseNet and long-short term memory networks (LSTM) to exploit the dependencies between abnormalities. Several authors also have worked on pneumonia classification. Khatri et al. [35] proposed the use of EMD (earth mover’s distance) to identify infected pneumonia lungs from normal non-infected lungs. Rahib et al. [36] and Okeke et al. [37] used a CNN model for pneumonia classification. Some researchers have shown assuring results such as Cohen et al. [38] and Rajaraman et al. [39]. Rajaraman et al. [39] tried to explain the performance of customized CNNs to detect pneumonia and further differentiate between bacterial and viral types in pediatric CXRs. Sirazitdinov et al. [40] used a region based convolutional neural network for segmenting the pulmonary images along with image augmentation for pneumonia identification. Lakhani and Sundaram [41] used the AlexNet and GoogLeNet neural networks with data augmentation and without any pre-training to obtain an area under the curve (AUC) of 0.94–0.95. Rajpurkar et al. [42] used CheXNeXt, a very deep CNN with 121 layers, to detect 14 different pathologies, including pneumonia, in frontal-view chest X-rays. A localization approach based on pre-trained DenseNet-121, along with feature extraction, was used to identify 14 thoracic diseases in [43]. Saraiva et al. [44], Ayan et al. [45], and Rahman et al. [46] used deep learning based methods for pneumonia classification. Xiao et al. [47] proposed a novel multi-scale heterogeneous three-dimensional (3D) convolutional neural network (MSH-CNN) based on chest computed tomography (CT) images. Xu et al. [48] used a hierarchical convolutional neural network (CNN) structure and a novel loss function, sin-loss, for pneumonia detection. Jaiswal et al. [49] used Mask-RCNN, utilizing both global and local features for pulmonary image segmentation, with dropout and L2 regularization, for pneumonia identification. Jung et al. [50] used a 3D deep CNN (3D DCNN), which had shortcut connections. Vikash et al. [51] combined the outputs of different neural networks and reached the final prediction using majority voting. None of the above-mentioned approaches except that of Vikash et al. [51] tried to combine predictions from different neural networks.

The main contribution is a weighted classifier that integrates five deep learning models. The weights for each model are based on each model’s performance on the testing dataset.

This paper is structured as follows: Section 3 deals with the methods used. A brief description of the methods used in this paper is given. The experimental dataset is introduced in Section 4. In Section 5, and the proposed methodology is discussed. In Section 6, the results obtained are discussed concerning different parameters. This section is followed by Section 7, containing the discussion, and Section 8, containing the conclusion of the paper.

## 3. Background of Deep Learning Methods

### 3.1. Convolutional Neural Network

LeCun et al. [52] first used CNN, in 1989, for handwritten zip code recognition. This is a type of feed-forward network. The main advantage of CNN compared to its predecessors is that it is capable of detecting the relevant features without any human supervision.

**Figure 1:**
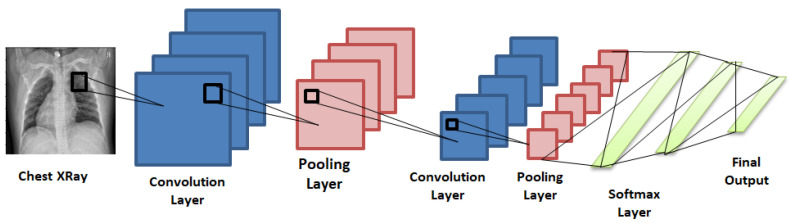
Convolutional neural network consisting of convolution and pooling layers and fully connected Softmax layers at the end to give the final prediction.

A series of convolution and pooling operations is performed on the input image, which is followed by a single or multiple fully connected layers, as shown in Figure 1. The output layer depends on the operations being performed. For multiclass classification, the output layer is a softmax layer. The main disadvantage with deeper CNNs is vanishing gradients, which can be solved by using residual networks introduced in the following section.

### 3.2. Transfer Learning

In transfer learning, a model that is trained for a particular task is employed as the starting point for solving another task. Therefore, in transfer learning, pre-trained models are used as the starting point for some specific tasks, instead of going through the long process of training with randomly initialized weights. Hence, it helps with saving the substantial computer resources needed to develop neural network models to solve these problems.

Pan and Yang [53] used domain, task, and marginal probabilities to propose a framework for better understanding the transfer learning. The domain *D* was defined as a two-element tuple consisting of the feature space, χ, with a marginal probability, P(X), where *X* is a sample data point. Hence, mathematically, domain *D* can be defined as,

(1) D={χ,P(X)}

Here, χ is the space of all term vectors, $x_{i}$ is the $i^{\mathrm{th}}$ term vector corresponding to some documents, and *X* is a particular learning sample $\left(X=x_{1},\cdots ,x_{n},\in \chi \right)$.

For a given domain *D*, the given task *T* is defined as:

(2) $$T=\left\{\gamma ,P\left(Y|X\right)\right\}=\left\{\gamma ,\eta \right\},Y=\left\{y_{1},\cdots ,y_{n}\right\},y_{i}\in \gamma$$

where γ is the label space. η is a predictive function learned from the feature vector/label pairs ($x_{i},y_{i}$), where $x_{i}\in \chi$ and $y_{i}\in \gamma$.

(3) $$\eta \left(x_{i}\right)=y_{i}$$

Here, η predicts a label for each feature vector.

Due to the lack of a sufficient dataset, training a deep learning based model for medical diagnosis related problems is computationally expensive, and the results achieved are also not up to the mark. Hence, pre-trained deep learning models, which were previously trained on ImageNet [54] dataset, were used in this paper. Further, all these pre-trained models were fine-tuned for pneumonia classification. All the layers of the architectures used were trainable. Further details, related to fine-tuning, are discussed in Section 5.2.

### 3.3. Pre-Trained Neural Networks

Five state-of-the-art deep learning networks, ResNet18, DenseNet121, InceptionV3, Xception, and MobileNetV2, were used in this study. They are briefly discussed in Appendix A at the end of the paper.

### 3.4. Performance Metrics for Classification

All the models were tested on the test dataset after the completion of the training phase. Their performance was validated using the accuracy, recall, precision, F1, and area under the curve (AUC) score. All the performance metrics used in this paper are discussed below.

In the below-mentioned definitions and equations, while classifying healthy and pneumonia patients, true positive (TP) denotes the number of pneumonia images identified as pneumonia, true negative (TN) denotes the number of normal images identified as normal (healthy), false positive (FP) denotes the number of normal images incorrectly identified as pneumonia images, and false negative (FN) denotes the number of pneumonia images incorrectly identified as normal.

- Accuracy: It tells us how close the measured value is to a known value.
  (4) $$Accuracy=\frac{\left(TP+FN\right)}{\left(TP+TF+FP+FN\right)}$$
- Precision: It tells about how accurate the model is in terms of those which were predicted positive.
  (5) $$Precision=\frac{TP}{\left(TP+FP\right)}$$
- Recall: It calculates the number of actual positives the model was able to capture after labeling it as positive (true positive).
  (6) $$Recall=\frac{TP}{\left(TP+FN\right)}$$
- F1: It gives a balance between precision and recall.
  (7) $$F1=2\times \frac{\left(Precision\times Recall\right)}{\left(Precision+Recall\right)}$$
- AUC Score and ROC Curve: ROC (receiver operating characteristics) is a probability curve, and AUC (area under curve) represents the degree of separability. The ROC curve is the plot of sensitivity (true positive rate) against specificity (false positive rate).

## 4. Materials

### Experimental Dataset

**Figure 2:**
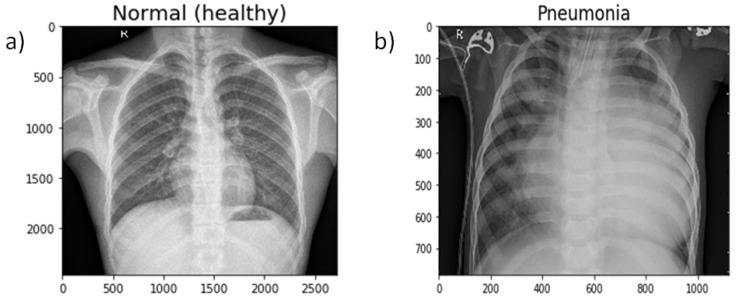
Chest Xray of (**a**) a healthy person and (**b**) a person suffering from pneumonia.

The dataset [55] comprised a total of 5836 images (Table 1) segmented into two main parts, a training set and a test set. Both bacterial and viral pneumonia were considered as a single category, pneumonia infected. The dataset used in this study did not include any case of viral and bacterial co-infection. All chest X-ray images were taken during the routine clinical care of the patients. Two expert physicians then graded the diagnoses for the images before being cleared for training the AI system. The evaluation set was also checked by a third expert to account for any grading errors. The proportion of data assigned to training and testing was highly imbalanced. Therefore, the dataset was shuffled and arranged into training and test sets only. Finally, there were 5136 images in the training set and 700 images in the test set. Eleven-point-nine-five percent of the complete dataset was used as the testing dataset. Figure 2 shows two chest X-ray images, one of a healthy person and the other of a person suffering from pneumonia.

## 5. Proposed Methodology

**Figure 3:**
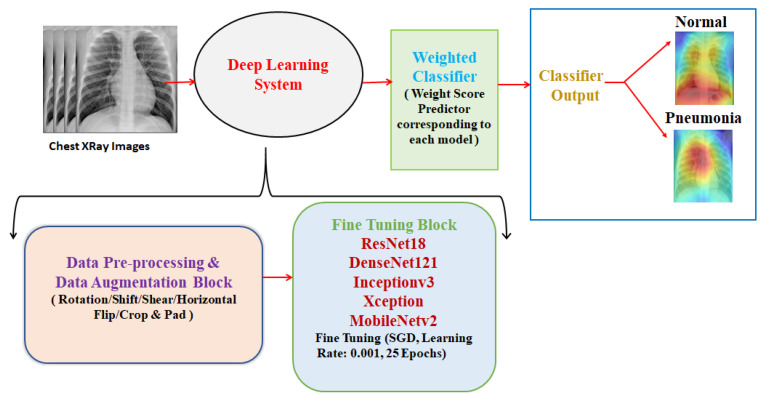
Block diagram of the proposed methodology (deep learning system consists of the data pre-processing and data augmentation block and the fine-tuning block; the weighted classifier gives the final prediction).

An optimum solution for the detection of pneumonia from chest X-rays is proposed in this paper. Data augmentation was used to address the problem of the limited dataset, and then, state-of-the-art deep learning models, as discussed in Section 3, were fine-tuned for pneumonia classification. Then, predictions from these models were combined, using a weighted classifier (discussed afterward in this section), to compute the final prediction. The complete block diagram of the proposed methodology can be seen in Figure 3.

### 5.1. Data Preprocessing and Augmentation

Each image had to be preprocessed according to the deep neural network used. There were two important steps involved: resizing and normalization. Different neural networks require images of different sizes according to their defined architecture. ResNet18, DenseNet121, and MobileNetV2 expect images of size 224 × 224, while InceptionV3 and Xception require images of size 229 × 229. All the images were also normalized according to the respective architectures.

Adequate training of a neural net requires big data. With less data availability, parameters are undermined, and learned networks generalize poorly. Data augmentation solves this problem by utilizing existing data more efficiently. It aids in increasing the size of the existing training dataset and helps the model not to overfit this dataset. In this case, there were a total of 1283 images of the normal (healthy) case and 3873 images of the pneumonia case in the training dataset. Out of these, four-hundred images were reserved for optimizing the weighted classifier. This dataset was highly imbalanced. There were already enough images in the pneumonia case. Therefore, each image of only the normal (healthy) case was augmented twice. Finally, after augmentation, there were 3399 healthy chest X-ray images and 3623 pneumonia chest X-ray images.

**Figure 4:**
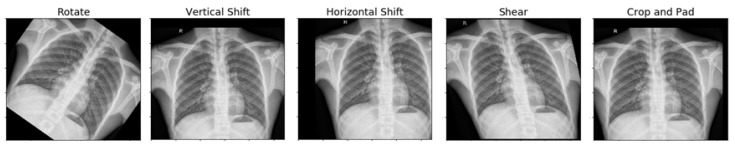
Resultant image after performing the augmentation technique.

The settings utilized in image augmentation are shown below in Table 2. The images after performing various augmentation techniques are shown below (Figure 4). Only one of these techniques was used to generate the augmented image.

### 5.2. Fine-Tuning the Architectures

All the architecture details used in this paper are discussed in Appendix A. Raw chest X-ray images, after being pre-processed and normalized, were used to train the network. Then, data augmentation techniques were used to process the dataset more efficiently. All the layers of the networks used were trainable, and these layers extracted the features from the images. Some parameters must be set to train the network. An interesting paper from UC Berkeley [56] came out, and according to it, stochastic gradient descent (SGD) had better generalization than adaptive optimizers. Therefore, SGD as the optimizer was used, and the model was trained for 25 epochs. The learning rate, the momentum, and the weight decay were set to 0.001, 0.9, and 0.0001, respectively (Table 3). These configurations were to make sure that the networks were fine-tuned for pneumonia diagnosis.

### 5.3. Weighted Classifier

In this module of the proposed methodology, a weight $\left(W_{k}\right)$ corresponding to each model was estimated. $W_{k}$ can be defined as the belief in the $k^{\mathrm{th}}$ model, with *k* being equal to 5 as 5 pre-trained models were used in this paper. $W_{k}$ has values between 0 and 1, and the sum of all weights is 1 (Equation (9)). Each model, after it was fine-tuned, returned the probabilities for each class label, i.e., 2 classes in the form of a matrix $\left(P_{k}\right)$. A weighted sum of all these predictions arrays was calculated (Equation (8)).

(8) $$P_{1}W_{1}+P_{2}W_{2}+P_{3}W_{3}+\cdots +P_{k}W_{k}=P_{f}$$

(9) $$W_{1}+W_{2}+W_{3}+\cdots +W_{k}=1$$

(10) $$Loss=-\frac{1}{N}\sum_{i=1}^{n}y\times log\left(p\right)+\left(1-y\right)\times log\left(1-p\right)$$

**Figure 5:**
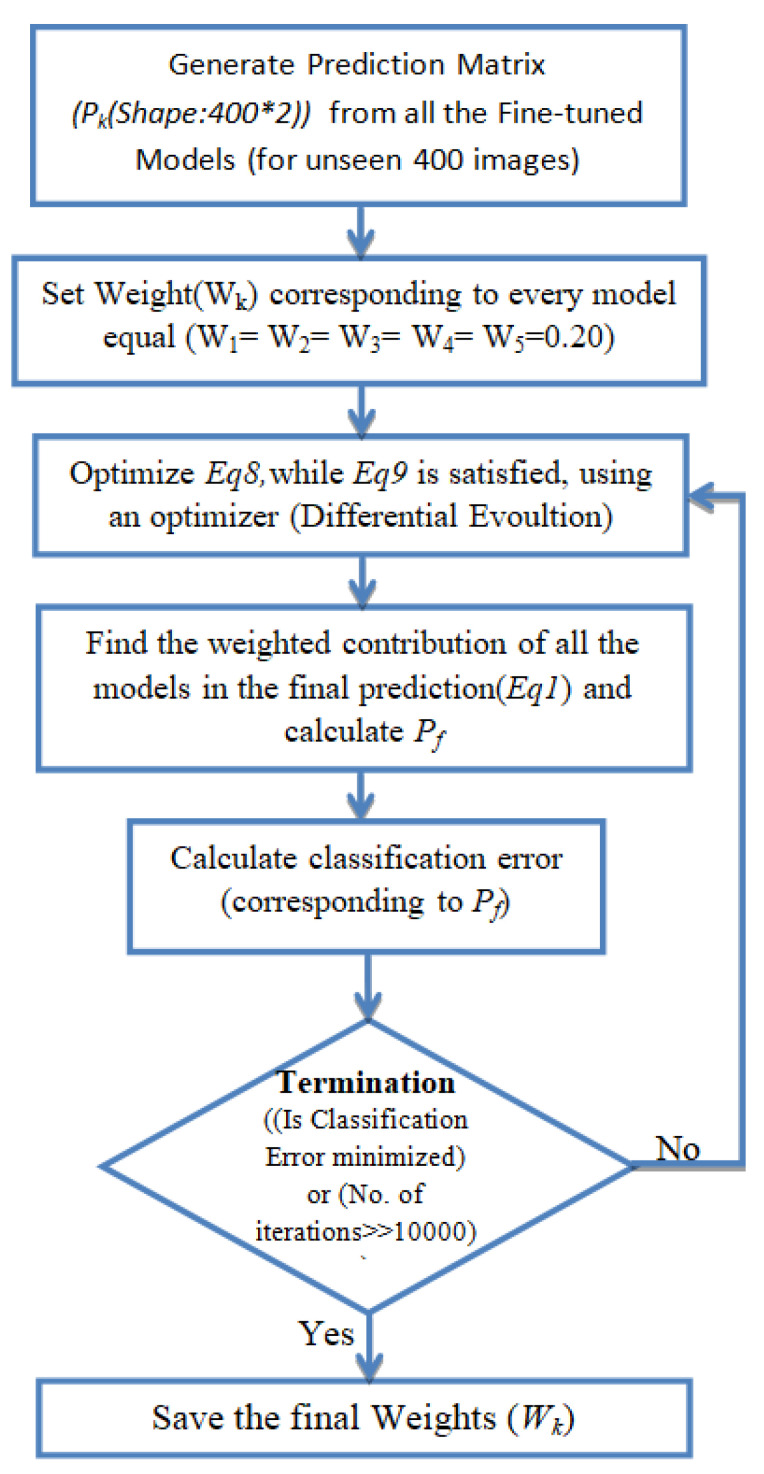
Procedure followed while calculating the optimal weight corresponding to every model.

**Figure 6:**
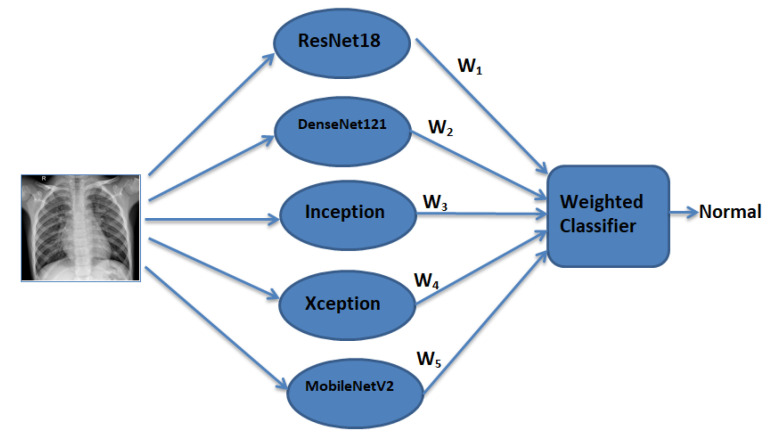
Weighted classifier module used in this paper (weighted predictions from all the models are passed to the weighted classifier, which gives the final weighted prediction).

$P_{k}$ is the prediction matrix, with shape: number of optimization images * class labels (400*2), corresponding to each architecture. In Equation (8), the contribution of each model is weighted by a coefficient $\left(W_{k}\right)$, which indicates the trust in the model. First, we obtained the $P_{k}$ for every model for an unseen image set (400 images). Then, Equation (8) was optimized such that the classification error was minimized and Equation (9) was also satisfied. We used differential evolution [57] for global optimization of Equation (8). Differential evolution is a stochastic global search algorithm. It optimized Equation (8) by iteratively refining a candidate solution with regard to Equation (9). Hence, optimizing Equation (8) would provide the $W_{k}$ values corresponding to each model. The value of $W_{k}$ for the $k^{\mathrm{th}}$ model depended on the respective models’ performance on the test dataset. The maximum iterations for differential evolution algorithms were kept to be 1000. With the help of $P_{f}$, the prediction of a class label could be computed. Classification loss corresponding to this $P_{f}$ was reduced while optimizing Equation (8). Log loss (Equation (10), also known as logistic loss or cross-entropy loss, was used as the loss function. In Equation (10), *N* denotes the size of the image set (400) and *p* denotes the probability that the given image is pneumonia infected. Figure 5 shows the process followed to find the optimal weight corresponding to each model. Figure 6 shows the weighted classifier used in the proposed methodology.

### 5.4. Class Activation Maps

**Figure 7:**
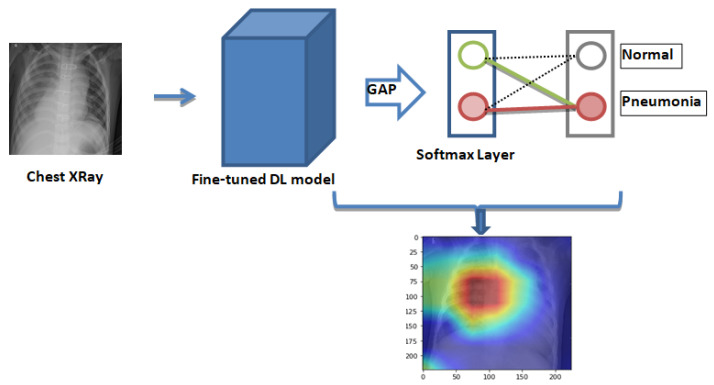
Generation of the class activation map for fine-tuned deep learning models. The layers of deep learning models are followed by the global average pooling layer (GAP) (⇒) and the softmax layer to give the final prediction. Features that are used for pneumonia detection get highlighted in the class activation map.

Class activation maps (CAMs) [58] can help in demystifying the results of deep learning models. Traditionally, deep learning based methods are considered to be a black-box approach. For clinical decision making, it is necessary that the results of the deep learning model can be interpreted. CAMs can help in identifying the parts of the image on which the model was focusing while making the final prediction and hence can provide insights into the working of the model. Such an analysis can further help in hyperparameter tuning and gain understanding of the reason behind the failure of the model. For obtaining the class activation map, the network needed to be trained with the global average pooling (GAP) layer. After the GAP layer, a fully connected network was maintained, which was followed by the softmax layer, providing the class, such as pneumonia, as shown in Figure 7. CAMS class activation maps were generated for both bacterial and viral pneumonia for all the fine-tuned model and are discussed in detail in the results section.

## 6. Experimental Results

In this section, the experiments and evaluation techniques used in the paper to test the efficiency of the proposed model are presented. The chest X-ray image dataset, proposed in [55], was used. The Keras open-source deep learning framework with TensorFlow as the backend was used, first to load the pre-trained architectures on the ImageNet Dataset [54] and then fine-tune them for the task at hand. All the computation work was done on a Standard PC with8 GB RAM, NVIDIA GeForce GTX 1060 6 GB GPU, and Intel i7, seventh-generation processor.

### 6.1. Result in Terms of Testing Accuracy and Testing Loss

**Figure 8:**
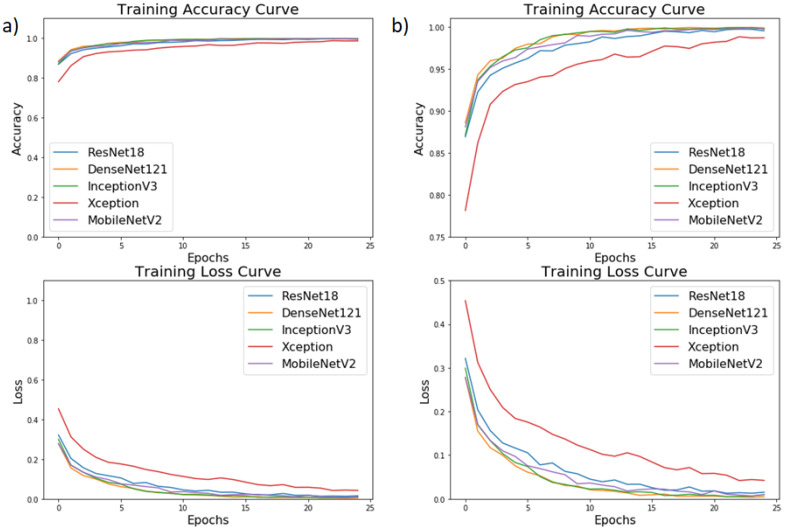
(**a**) Training accuracy and training loss curves for different architectures over the training dataset while the models were trained for 25 epochs, (**b**) Zoom-in version of (**a**).

To test and evaluate the performance of the proposed network, each experiment was conducted five times. Parameters and hyperparameters were tuned during the training. Figure 8 shows the training accuracy and training loss curves obtained while training the models for 25 epochs. The training accuracy for all the models exceeded 99%, and the training loss for all the models was below 0.03. Except for Xception, all the other models had similar training accuracy and training loss curves. Table 4 summarizes the testing accuracy and testing loss for different networks and the final weighted classifier. DenseNet121 was able to attain the maximum testing accuracy and the minimum testing loss. Initially, all the weights of the weighted classifier were kept equal $\left(W_{1}=W_{2}\cdots W_{5}=0.20\right)$. Hence, every model contributed equally towards the final prediction. A test accuracy of 97.45 and a loss of 0.087 was obtained. Then, the optimum weights were estimated for every model. The value of these estimated weights is shown in Table 5. With these weights, the final weighted classifier was able to achieve a testing accuracy of 98.43, and the testing loss was 0.062.

In Table 4, it can be seen that when equal weights were assigned to every model, the testing accuracy of the weighted classifier was less than that of DenseNet121. This could be attributed to the fact that even the models with less testing accuracy were assigned the same weights as that to the models with higher testing accuracy. Finally, when optimum weights were calculated, the testing accuracy of the weighted classifier showed an improvement of 0.98%. Table 5 shows that the weight assigned to every model depended on its performance on the test dataset. Hence, it could be said that the weight assigned to a model represented the belief or trust in that model. The maximum weight was assigned to DenseNet121, which was followed by ResNet18.

All the test images were pre-processed similarly as the training images and hence had the same size as required by the respective architecture. The test images were of size 224 × 224 for ResNet18, DenseNet121, and MobileNetV2, while for InceptionV3 and Xception, they were of size 229 × 229. The testing was also done on the same system on which training was done. The average inference time for all the models was 0.045 s (while the GPU was used), and for the weighted classifier, it was 0.203 s.

### 6.2. Performance Analysis

**Figure 9:**
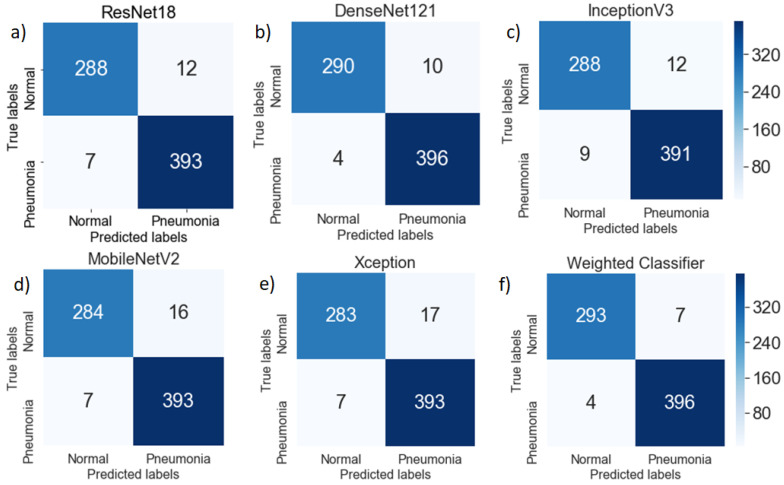
Confusion matrix for (**a**) ResNet18, (**b**) DenseNet121, (**c**) InceptionV3, (**d**) MobileNetV2, (**e**) Xception, and (**f**) Weighted Classifier architectures and the weighted classifier over the testing dataset. False positives were greater than the false negatives for all the models.

To further test the robustness of the proposed methodology, the accuracy, recall, precision score, F1 score, and AUC score for all the models and the proposed weighted classifier were calculated. To calculate the mentioned scores, confusion matrices for all the architectures were obtained (Figure 9). With the help of the confusion matrix, the number of true positives, true negatives, false positives, and false negatives could be calculated, which further helped in checking the efficacy of the model.

As the recall was increased, the precision decreased, and vice versa. In medical applications, all the patients who had the disease needed to be identified, and hence, the recall could be maximized. A low recall could be accepted if the cost of a follow-up medical examination was not high. Hence, the F1 score could be used to find the optimal blend of precision and recall.

In the plotted confusion matrices (Figure 9), it can be seen that the proposed weighted classifier outperformed all the individual models. The generic image features, learned by the deep learning models from ImageNet, served as a good initialization of the weights. The misclassification error for normal (healthy) images as pneumonia images was greater compared to pneumonia images as healthy images. This might be because the number of chest X-ray images of the normal (healthy) case was significantly lower compared to the pneumonia cases.

**Figure 10:**
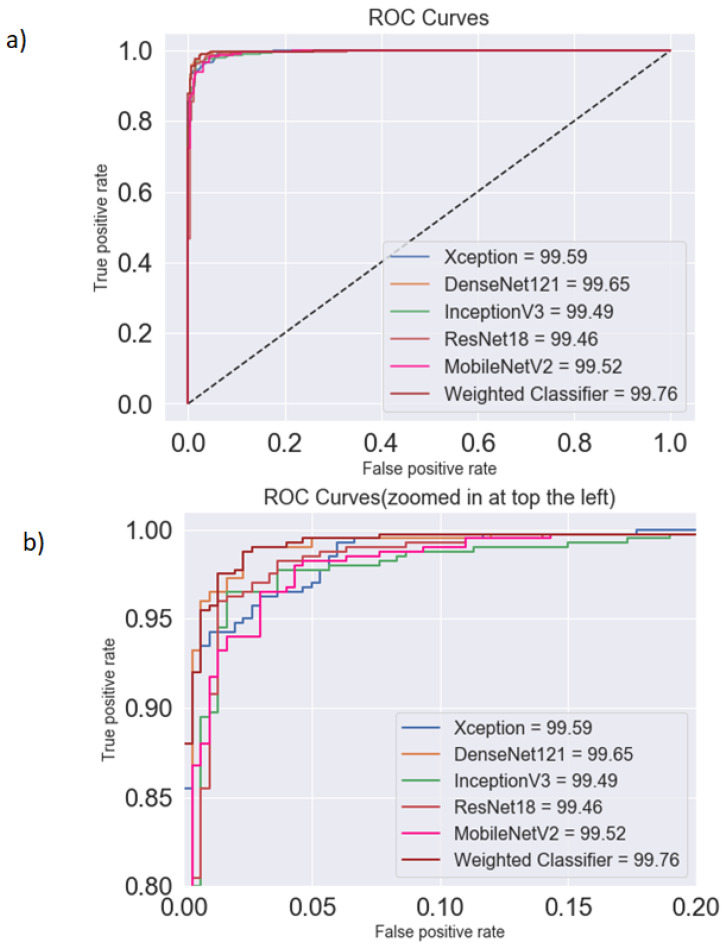
Comparison between (**a**) the AUC (area under the curve) and (**b**) ROC (receiver operating characteristics) curves for different architectures and the weighted classifier for the testing dataset. The weighted classifier (brown line), followed by DenseNet121 (yellow line), had the highest AUC.

Figure 10 shows the ROC curves for different architectures and the proposed classifier. The maximum AUC score (99.76) was achieved by the proposed classifier. All the models had a similar AUC/ROC curve. All the results are tabulated in Table 6. After analyzing the results, it can be said the weighted classifier gave the best results with an AUC score of 99.75, F1 score of 98.63, and test accuracy of 98.43. Hence, the proposed weighted classifier was able to combine the predictions from all the individual architectures in an optimum manner. The differences in the performance of other models were not significant. This might be because all the models used in this paper were deep learning based and were fine tuned on the same insufficient dataset.

### 6.3. Explanation of the Results Using Heat Maps

**Figure 11:**
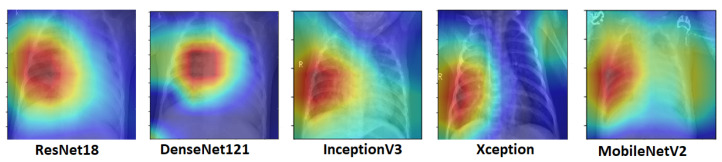
Activation maps for chest X-rays having bacterial pneumonia corresponding to different architectures. Abnormal lungs, in the case of bacterial pneumonia, were detected by the deep learning models.

**Figure 12:**
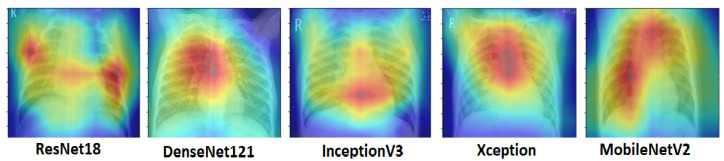
Activation maps for chest X-rays having viral pneumonia corresponding to different architectures. Viral pneumonia, with a more diffused “interstitial” pattern in both lungs, was detected by the deep learning models.

The activation maps were plotted for every individual network. These activation maps helped in localizing areas in the image most indicative of pneumonia. The activation maps were obtained for the last convolutional layer of each network. In the case of bacterial pneumonia (Figure 11), all the networks detected the abnormal lung to predict the presence of pneumonia correctly. Viral pneumonia manifested with a more diffuse “interstitial” pattern in both lungs, which was detected by all the fine-tuned architectures [59] (Figure 12).

### 6.4. Comparative Analysis of Various Existing Methods

The accuracy of various existing methods and the proposed methodology were compared. All the results mentioned in this section are reported by the authors in their respective studies. Rahib H. Abiyey et al. [36] used CNN and achieved a validation accuracy of 92.4%. The test dataset used was smaller compared to this paper. Okeke Stephen et al. [37] achieved a validation accuracy of 93.73% with their own CNN model. No other metric was published in either of these works. Cohen et al. [38] used a model based on DenseNet-121. They reported an AUC score of 98.4%. Unfortunately, the other metrics were not reported in the paper. Rajaraman et al. [39] used customized CNNs to detect pneumonia and reported a test accuracy of 96.2%. M.Togacar et al. [60] combined features from different deep learning models for pneumonia classification and achieved an accuracy of 96.84%. Vikash et al. [51] combined the outputs of different neural networks and reached the final prediction using majority voting. They achieved an AUC score of 99.34. Saraiva et al. [44], Ayan et al. [45], and Rahman et al. [46] used deep learning based methods and achieved an accuracy of 94.4%, 84.5%, and 98.0%, respectively. In all of these papers, the dataset used was of a similar size. All the studies other than Rahib H.Abiyey et al. [36] used image augmentation techniques. All the above-discussed results are summarized in Table 7.

It can be seen in the above table that the proposed methodology surpassed the other approaches in terms of precision, recall, accuracy, and the AUC score. The weighted classifier showed the best accuracy of all the recent studies to the best of our knowledge.

## 7. Discussion

The high test accuracy (98.43) and AUC score (99.76) showed that the proposed method could be used as a supplement in clinical decision making. It can only aid the radiologists in the decision making process; the final decision has to be made by an expert. The proposed weighted classifier, with optimum weights, showed an improvement of 0.98%, in terms of the testing accuracy, over the case in which equal weights were assigned to every model. The false positives were greater than the false negatives, and hence, the classification error of pneumonia suffering patients as healthy was comparatively lesser, which is ideally required in medical diagnosis. Further, the activation maps plotted in this paper showed that the deep learning based models used were able to identify pneumonia affected regions in the chest X-rays. When compared to DenseNet121, the proposed weighted classifier showed an improvement of 0.43% in terms of testing accuracy, which in the real world on a large test dataset would be a significant number.

One of the limitations of this approach was the scarcity of available data. Usually, deep learning models are trained over thousands of images. Training deep neural networks with limited data might lead to overfitting and restricts the models’ generalization ability. Unlike large datasets like ImageNet, the variability in the chest X-ray data was several orders of magnitude smaller. The performance of the proposed methodology would only increase with the availability of more data.

Another limitation was that the results of the deep learning models could not be properly explained. A deep understanding of the radiological features visible in chest X-rays is required for the diagnosis of the disease from the X-rays. The proper explanation of the final prediction of the model is also required, and this is one of the drawbacks of the deep learning based models. To this end, the activation maps were plotted, but further work is required. In the future, with better annotated datasets available, deep learning based methods might be able to solve this problem.

## 8. Conclusions and Future Scope

Pneumonia constitutes a significant cause of morbidity and mortality. It accounts for a considerable number of adult hospital admissions, and a significant number of those patients ultimately die (with a mortality rate of 24.8% for patients over 75 years) [61]. According to the WHO, pneumonia can be prevented with a simple intervention and early diagnosis and treatment [4]. Nevertheless, the majority of the global population lacks access to radiology diagnostics [62]. Even when there is the availability of imaging equipment, there is a shortage of experts who can examine X-rays. Through this paper, the automatic detection of pneumonia in chest X-ray images using deep transfer learning techniques was proposed. The deep networks, which were used in our methodology, had more complex structures, but fewer parameters and, hence, required less computation power, but achieved higher accuracy. Transfer learning and data augmentation were used to solve the problem of overfitting, which is seen when there is insufficient training data, as in the case of medical image processing. Further, to combine different architectures efficiently, a weighted classifier was proposed. The experiments were performed, and the different scores obtained, such as the accuracy, recall, precision, and AUC score, proved the robustness of the model. The proposed model was able to achieve an accuracy of 98.857%, and further, a high F1 score of 99.002 and AUC score of 99.809 affirmed the efficacy of the proposed model. Though many methods have been developed to work on this dataset, the proposed methodology achieved better results. In the future, it would be interesting to see approaches in which the weights corresponding to different models can be estimated more efficiently and a model that takes into account the patient’s history while making predictions.

## Figures and Tables

**Table 1:** Description of the experimental dataset.

Category	Training Set	Test Set
Normal (Healthy)	1283	300
Pneumonia (Viral + Bacteria)	3873	400
Total	5156	700
Percentage	88.05%	11.95%

**Table 2:** Augmentation techniques used in the proposed methodology.

Technique	Setting
Rotation	45
Vertical Shift	0.2
Horizontal Shift	0.15
Shear	16
Crop and Pad	0.25

**Table 3:** Hyper-parameters used while fine-tuning the deep learning models.

Architecture	Image Size	Epochs	Optimizer	Learning Rate	Momentum	Weight Decay
ResNet18	224 × 224					
DenseNet121	224 × 224					
InceptionV3	229 × 229	25	Stochastic Gradient Descent	0.001	0.9	0.0001
Xception	229 × 229					
MobileNetV2	224 × 224					

**Table 4:** Final testing accuracy and testing loss achieved by all the architectures and the weighted classifier.

Architecture	Testing Accuracy	Testing Loss
ResNet18	97.29	0.096
DenseNet121	98.00	0.064
Inception	97.00	0.098
Xception	96.57	0.101
MobileNetV2	96.71	0.096
Weighted Classifier (With Equal Weights)	97.45	0.087
Weighted Classifier (With Optimized Weights)	98.43	0.062

**Table 5:** Weight value (belief or trust value) corresponding to every architecture.

Architecture	Weight
ResNet18 (W1)	0.25
DenseNet121 (W2)	0.30
Inception (W3)	0.18
Xception (W4)	0.08
MobileNetV2 (W5)	0.19

**Table 6:** Accuracy, precision, recall, F1 score, and AUC score corresponding to different architectures.

Architecture	Accuracy	Precision	Recall	F1 Score	AUC Score
ResNet18	97.29	97.03	98.25	97.63	99.46
DenseNet121	98.00	97.53	99.00	98.26	99.65
InceptionV3	97.00	97.02	97.75	97.39	99.49
Xception	96.57	95.85	98.25	97.03	99.59
MobileNetV2	96.71	96.08	98.25	97.15	99.52
Weighted Classifier	98.43	98.26	99.00	98.63	99.76

**Table 7:** Comparison of the proposed methodology with different existing methods.

Model	No. of Images	Precision	Recall	Accuracy	AUC
Rahib H.Abiyey et al. [36]	1000	-	-	92.4	-
Okeke Stephen et al. [37]	5856	-	-	93.73	-
Cohen et al. [38]	5232	90.1	93.2	92.8	99.0
Rajaraman et al. [39]	5856	97.0	99.5	96.2	99.0
M.Togacar et al. [60]	5849	96.88	96.83	96.84	96.80
Saraiva et al. [44]	5840	94.3	94.5	94.4	94.5
Ayan et al. [45]	5856	91.3	89.1	84.5	87.0
Rahman et al. [46]	5247	97.0	99.0	98.0	98.0
Vikash et al. [51]	5232	93.28	**99.6**	96.39	99.34
Proposed Methodology	**5856**	**98.26**	99.00	**98.43**	**99.76**

The numbers are bold to show the best performance.

**Figure A1:**
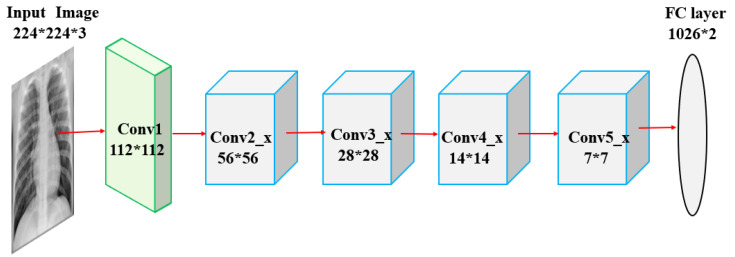
ResNet18 architecture comprised of a convolutional (112*112) layer at the beginning and five convolutional blocks.

**Figure A2:**
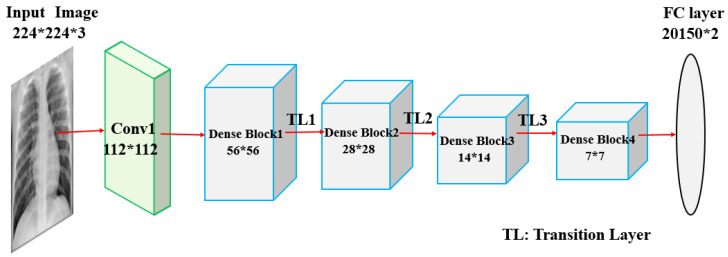
Densenet121 architecture with the convolutional (112*112) layer at the beginning and five dense blocks.

**Figure A3:**
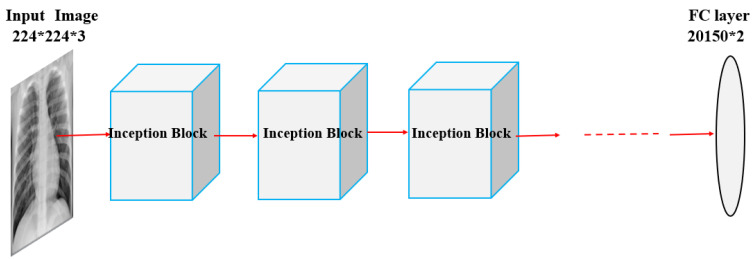
InceptionV3 architecture with Inception blocks comprising 1*1, 3*3, and 5*5 convolutions.

**Figure A4:**
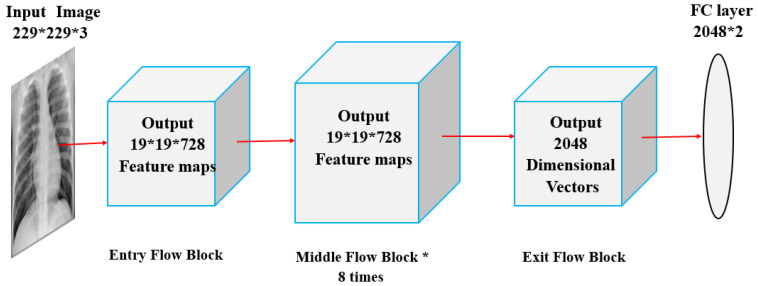
Xception architecture divided into three blocks: entry flow block, middle flow block, and exit flow block.

**Figure A5:**
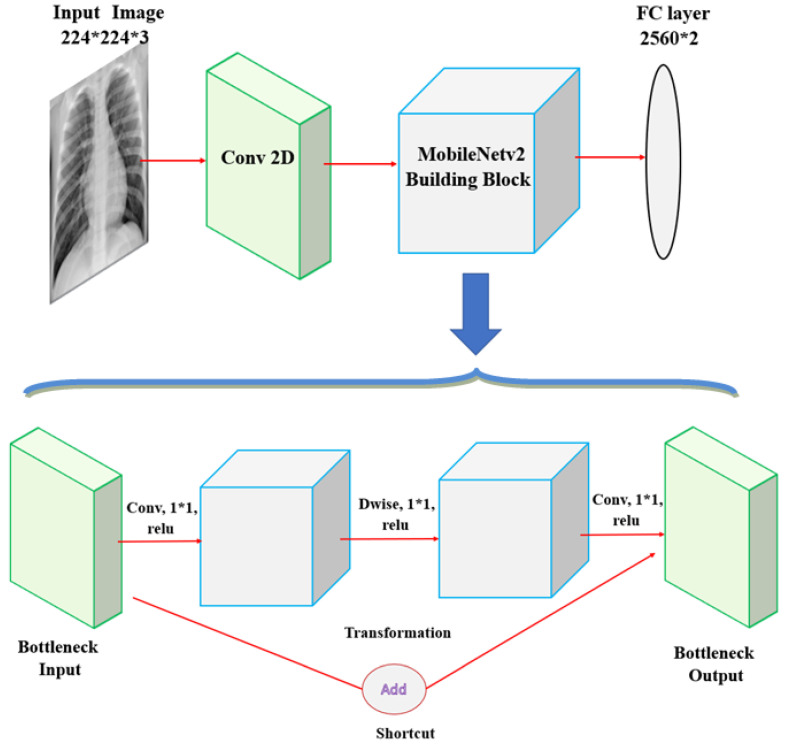
MobileNetV2 architecture with the MobileNetv2 building block comprising bottlenecks and depth-wise separable convolutions.

## Abbreviations

The following abbreviations are used in this manuscript:

2D	2-dimensional
3D	3-dimensional
AI	Artificial intelligence
AUC	Area under the curve
BPNN	Back propagation neural network
CNN	Convolutional neural network
CpNN	Competitive neural network
CT	Computed tomography
DDR	Double data rate
GPU	General processing unit
DNN	Deep neural network
PC	Personal computer
SGD	Stochastic gradient descent
UNICEF	United Nations Children’s Fund
WHO	World Health Organization

## Appendix A. Pre-Trained Neural Networks Used in the Paper

### Appendix A.1. ResNet18

With deeper neural networks, the degradation problem is exposed, and with network depth increasing, accuracy gets saturated and then degrades rapidly. Kaiming He et al. first proposed residual networks at the Microsoft Research Institute [63] to solve this problem. They explained the architecture of residual networks in their paper. Here, the incoming eigenvalues are processed by both the network parameter layers, and their original values are also taken into account as a part of the output obtaining the result. Bypass connections are an integral part of the architecture and can skip one or more layers. Here, bypass connections are used to implement identity mapping, and their outputs are summed with the outputs of the stacked layers. The complete network consists of five convolution blocks and can be easily trained using SGD with backpropagation. Lastly, Figure A1 shows the ResNet18 architecture used in the paper.

### Appendix A.2. DenseNet121

Studies have shown that the connections between layers close to the input and those close to the output help in increasing the performance of convolutional networks. This idea was implemented in residual networks [63] and also in dense convolutional networks (DenseNet) [64]. DenseNet requires lesser parameters compared to conventional convolutional networks as it does not require relearning excessive feature maps because of its dense connectivity pattern. The network is divided into dense blocks, with the dimensions of the feature maps remaining constant within a block and the number of filters changing between them. DenseNet has several advantages such as the number of parameters is significantly reduced, the features are reused, and the vanishing gradient is mitigated. Figure A2 depicts the pre-trained DenseNet architecture used in the paper.

### Appendix A.3. InceptionV3

The bigger the deep learning model is, the more prone it is to overfitting, and increasing the number of parameters also means the need to increase the computational resources. In the Inception model [65] These problems are addressed, which allows increasing the depth and width of the deep learning model and also keeping the computational cost constant. A sparsely connected network architecture is introduced in this model. One by one, 3*3, and 5*5 convolutions are computed, and an auxiliary classifier is used as a regularizer in the architecture. With only 42 layers, it is more efficient compared to VGGNet [66]. This model was trained on the ImageNet dataset. Figure A3 shows the pre-trained InceptionV3 model used in this paper.

### Appendix A.4. Xception

The Xception model was proposed by Francois Chollet [67]. Xception is an extension of the Inception architecture where the standard Inception modules are replaced by modified depthwise separable convolutions, which makes it better for both the ImageNet ILSVRCand JFTdatasets. The modified depthwise separable convolution is the pointwise convolution, which is followed by a depthwise convolution. There are also residual/skip connections as proposed in the ResNet architecture [63]. The Xception architecture can be divided into three blocks: entry flow, middle flow, and exit flow. The middle flow block is repeated eight times. There is no intermediate non-linearity in modified depthwise separable convolution in the architecture. The pre-trained model of Xception used in the paper is shown in Figure A4.

### Appendix A.5. MobileNetV2

MobileNets are small and low power models developed by a group of researchers from Google [68]. MobileNetV2 is an upgrade to MobileNetV1 and uses depth-wise separable convolutions as efficient building blocks. There are two types of blocks in the architecture: one is a residual block with a stride of one, and the other one is a block with a stride of two for downsizing. Other than this, it has linear bottlenecks between the layers where the use of linear layers is essential, as it blocks nonlinearities from damaging too much information. These bottlenecks help the model in encoding the intermediate inputs and outputs. The inner layer helps in transforming lower-level concepts such as pixels to higher-level descriptors such as image categories. There are also shortcut connections between the bottlenecks. A pre-trained MobileNetV2 was used in this paper (Figure A5). Transfer learning was used to optimize the model for pneumonia classification.
